# Association between body mass index and subcortical brain volumes in bipolar disorders–ENIGMA study in 2735 individuals

**DOI:** 10.1038/s41380-021-01098-x

**Published:** 2021-04-16

**Authors:** Sean R. McWhinney, Christoph Abé, Martin Alda, Francesco Benedetti, Erlend Bøen, Caterina del Mar Bonnin, Tiana Borgers, Katharina Brosch, Erick J. Canales-Rodríguez, Dara M. Cannon, Udo Dannlowski, Ana M. Díaz-Zuluaga, Torbjørn Elvsåshagen, Lisa T. Eyler, Janice M. Fullerton, Jose M. Goikolea, Janik Goltermann, Dominik Grotegerd, Bartholomeus C. M. Haarman, Tim Hahn, Fleur M. Howells, Martin Ingvar, Tilo T. J. Kircher, Axel Krug, Rayus T. Kuplicki, Mikael Landén, Hannah Lemke, Benny Liberg, Carlos Lopez-Jaramillo, Ulrik F. Malt, Fiona M. Martyn, Elena Mazza, Colm McDonald, Genevieve McPhilemy, Sandra Meier, Susanne Meinert, Tina Meller, Elisa M. T. Melloni, Philip B. Mitchell, Leila Nabulsi, Igor Nenadic, Nils Opel, Roel A. Ophoff, Bronwyn J. Overs, Julia-Katharina Pfarr, Julian A. Pineda-Zapata, Edith Pomarol-Clotet, Joaquim Raduà, Jonathan Repple, Maike Richter, Kai G. Ringwald, Gloria Roberts, Raymond Salvador, Jonathan Savitz, Simon Schmitt, Peter R. Schofield, Kang Sim, Dan J. Stein, Frederike Stein, Henk S. Temmingh, Katharina Thiel, Neeltje E. M. van Haren, Holly Van Gestel, Cristian Vargas, Eduard Vieta, Annabel Vreeker, Lena Waltemate, Lakshmi N. Yatham, Christopher R. K. Ching, Ole Andreassen, Paul M. Thompson, Tomas Hajek, for the ENIGMA Bipolar Disorders Working Group

**Affiliations:** 1 Department of Psychiatry, Dalhousie University, Halifax, NS Canada; 2 Department of Clinical Neuroscience, Karolinska Institutet, Stockholm, Sweden; 3 Vita-Salute San Raffaele University, Milan, Italy; 4 Division of Neuroscience, Psychiatry and Psychobiology Unit, IRCCS San Raffaele Scientific Institute, Milan, Italy; 5 Unit for Psychosomatics / CL Outpatient Clinic for Adults, Division of Mental Health and Addiction, Oslo University Hospital, Oslo, Norway; 6 Institut d’Investigacions Biomèdiques August Pi i Sunyer (IDIBAPS), Centro de Investigación Biomédica en Red de Salud Mental (CIBERSAM), Barcelona Bipolar Disorders and Depressive Unit, Hospital Clinic, Institute of Neurosciences, University of Barcelona, Barcelona, Spain; 7 Department of Psychiatry, University of Münster, Münster, Germany; 8 Department of Psychiatry and Psychotherapy, Philipps-University Marburg, Marburg, Germany; 9 FIDMAG Germanes Hospitalàries Research Foundation, Barcelona, Spain; 10 Centre for Neuroimaging & Cognitive Genomics (NICOG), Clinical Neuroimaging Laboratory, NCBES Galway Neuroscience Centre, College of Medicine Nursing and Health Sciences, National University of Ireland Galway, Galway, Ireland; 11 Research Group in Psychiatry GIPSI, Department of Psychiatry, Faculty of Medicine, Universidad de Antioquia, Medellín, Colombia; 12 Norwegian Centre for Mental Disorders Research (NORMENT), Institute of Clinical Medicine, University of Oslo, Oslo, Norway; 13 Department of Neurology, Division of Clinical Neuroscience, Oslo University Hospital, Oslo, Norway; 14 Institute of Clinical Medicine, University of Oslo, Oslo, Norway; 15 Department of Psychiatry, University of California, San Diego, La Jolla, CA USA; 16 Desert-Pacific MIRECC, VA San Diego Healthcare, San Diego, CA USA; 17 Neuroscience Research Australia, Randwick, NSW Australia; 18 School of Medical Sciences, University of New South Wales, Sydney, NSW Australia; 19 Department of Psychiatry, University Medical Center Groningen, University of Groningen, Groningen, The Netherlands; 20 Neuroscience Institute, University of Cape Town, Cape Town, South Africa; 21 Department of Psychiatry and Mental Health, University of Cape Town, Cape Town, South Africa; 22 Department of Psychiatry and Psychotherapy, University of Bonn, Bonn, Germany; 23 Laureate Institute for Brain Research, Tulsa, OK USA; 24 Department of Neuroscience and Physiology, Sahlgrenska Academy at Gothenburg University, Gothenburg, Sweden; 25 Department of Medical Epidemiology and Biostatistics, Karolinska Institutet, Stockholm, Sweden; 26 Institute of Clinical Medicine, Department of Neurology, University of Oslo, Oslo, Norway; 27 Center for Mind, Brain and Behavior (CMBB), University of Marburg and Justus Liebig University Giessen, Marburg, Germany; 28 School of Psychiatry, University of New South Wales, Sydney, NSW Australia; 29 UCLA Center for Neurobehavioral Genetics, Los Angeles, CA USA; 30 Department of Psychiatry, Erasmus University Medical Center, Rotterdam, The Netherlands; 31 Research Group, Instituto de Alta Tecnología Médica, Ayudas diagnósticas SURA, Medellín, Colombia; 32 Institute of Psychiartry, King’s College Londen, London, UK; 33 Oxley College of Health Sciences, The University of Tulsa, Tulsa, OK USA; 34 West Region, Institute of Mental Health, Singapore, Singapore; 35 Yong Loo Lin School of Medicine, National University of Singapore, Singapore, Singapore; 36 South African MRC Unit on Risk & Resilience in Mental Disorders, University of Cape Town, Cape Town, South Africa; 37 Department of Child and Adolescent Psychiatry and Psychology, Erasmus University, Rotterdam, The Netherlands; 38 Department of Psychiatry, University Medical Center Utrecht Brain Center, University Medical Center Utrecht, Utrecht University, Utrecht, The Netherlands; 39 University of British Columbia, Vancouver, BC Canada; 40 Imaging Genetics Center, Mark and Mary Stevens Neuroimaging and Informatics Institute, Keck School of Medicine, University of Southern California, Marina del Rey, CA USA; 41 National Institute of Mental Health, Klecany, Czech Republic

**Keywords:** Bipolar disorder, Neuroscience

## Abstract

Individuals with bipolar disorders (BD) frequently suffer from obesity, which is often associated with neurostructural alterations. Yet, the effects of obesity on brain structure in BD are under-researched. We obtained MRI-derived brain subcortical volumes and body mass index (BMI) from 1134 BD and 1601 control individuals from 17 independent research sites within the ENIGMA-BD Working Group. We jointly modeled the effects of BD and BMI on subcortical volumes using mixed-effects modeling and tested for mediation of group differences by obesity using nonparametric bootstrapping. All models controlled for age, sex, hemisphere, total intracranial volume, and data collection site. Relative to controls, individuals with BD had significantly higher BMI, larger lateral ventricular volume, and smaller volumes of amygdala, hippocampus, pallidum, caudate, and thalamus. BMI was positively associated with ventricular and amygdala and negatively with pallidal volumes. When analyzed jointly, both BD and BMI remained associated with volumes of lateral ventricles  and amygdala. Adjusting for BMI decreased the BD vs control differences in ventricular volume. Specifically, 18.41% of the association between BD and ventricular volume was mediated by BMI (*Z* = 2.73, *p* = 0.006). BMI was associated with similar regional brain volumes as BD, including lateral ventricles, amygdala, and pallidum. Higher BMI may in part account for larger ventricles, one of the most replicated findings in BD. Comorbidity with obesity could explain why neurostructural alterations are more pronounced in some individuals with BD. Future prospective brain imaging studies should investigate whether obesity could be a modifiable risk factor for neuroprogression.

## Introduction

Bipolar disorders (BD) are among the most disabling and expensive psychiatric illnesses [1–3]. Yet, BD affects each person differently. Some individuals with BD show marked neuroimaging alterations, whereas the brains of others appear to be comparable to those of controls [4, 5]. Consequently, the strength and even the direction of associations between BD and individual brain imaging measures vary widely across studies [6–10]. We need to better understand why neurobiological findings differ within the same diagnosis and which factors underly this heterogeneity. One potential source of differences among individuals with BD is the comorbidity with medical conditions known to affect the brain [11]. One such condition, which targets the brain and is disproportionately frequent in BD, is obesity.

Between one-half and two-thirds of individuals with BD are overweight or obese. This represents a 1.6 times greater risk of obesity in BD than in the general population [12, 13]. Higher rates of obesity in BD may be related to shared genetics, pathophysiology, risk factors, including effects of medications or lifestyle factors [14, 15]. Regardless of the reasons for the comorbidity, obesity may be relevant for brain alterations in BD. The brain is now recognized as one of the targets for obesity-related damage [16–18]. Data from 12,087 individuals from the UK Biobank demonstrated that those with obesity had smaller volumes of several subcortical regions, including basal ganglia, hippocampus, and thalamus [19], which was in line with results from another large community-based sample [20]. Two meta-analyses also reported associations between measures of obesity and regional gray matter volumes, including hippocampus and temporal lobes [16, 21]. The same regions are often implicated in the neurobiology of BD [22] and show volumetric alterations in individuals with BD [23].

Whereas the negative association between obesity and cortical measures appears relatively uniform and replicated, there is less consistency in the direction and location of obesity-associated subcortical alterations [16, 19–21, 24]. Therefore, more research specifically focusing on obesity and subcortical regions is needed in general, but especially in psychiatric disorders. Relative to cortical measures, subcortical volumes are generally less linked to the genetic mechanisms of major psychiatric disorders [24–26]. Yet, subcortical changes are associated with BD [23] and are sensitive to other BD-related factors, such as medications [8] and metabolic alterations [19, 27]. Thus, we chose subcortical volumes as an initial dependent variable, to study the associations between extra-diagnostic factors and gray matter in BD. We hypothesized that obesity might help explain some of the subcortical brain changes in BD. Furthermore, the varying degrees of obesity may contribute to the varying degrees of brain alterations in people with the same diagnosis of BD, which are particularly pronounced in subcortical regions [8, 9, 23].

Despite the replicated associations between obesity and brain structure and the high prevalence of overweight/obesity in BD, the relationship of obesity to brain volume in BD remains underresearched. The available studies have focused on highly selected samples, i.e., individuals with the first episode of mania [28–30], adolescent BD participants [31], or offspring of people with BD [32]. The findings showed that in BD, elevated body mass index (BMI) was associated with brain structure, possibly with a stronger effect size or with some regional specificity compared to non-BD controls. In addition, obesity-related metabolic alterations were associated with subcortical regional volumes in BD [27], and obesity contributed to advanced brain age in first-episode psychosis [4]. Yet, many questions remain.

First, we should confirm the links between BMI and brain structure in larger, more generalizable samples of people with BD. Second, we need to better understand the interplay between BD and BMI. Are the associations between BD or BMI and brain structure specific to each factor, additive or is there an interaction? We could even be misinterpreting some for the brain changes as related to BD, when they may be better explained by obesity. Addressing these questions requires large samples, but it is crucial for the interpretation of brain imaging findings and for potential clinical translation. Such efforts could help us identify modifiable risk factors for brain alterations in BD, as well as provide insights into clinical heterogeneity and brain impacts of pharmacotherapy, which is often associated with weight gain [33, 34]. Thus, we jointly investigated the association between BD, BMI, and subcortical brain volumes in a large, highly generalizable, multicenter sample from the ENIGMA-BD working group.

## Methods

### Participating sites

The ENIGMA-BD Working Group brings together researchers with brain imaging and clinical data from people with BD [5, 23, 35, 36]. Seventeen site members of this group from 13 countries on 6 continents contributed individual subject structural MRI data, medication information, and BMI values from a total of 1134 individuals with BD and 1601 healthy controls. Based on previously reported effect sizes [23], this sample size was expected to provide adequate power (*n* = 233 per group minimum, power = 0.80, alpha = 0.05). Supplementary Tables S1 and S2 list the demographic/clinical details for each cohort. Supplementary Table S3 provides the diagnostic instruments used to obtain diagnosis and clinical information. Supplementary Table S4 lists exclusion criteria for study enrollment. Briefly, all studies used standard diagnostic instruments, including SCID (*N* = 12), MINI (*N* = 2), and DIGS (*N* = 1). Most studies (*N* = 10) included both bipolar I (BDI) and bipolar II (BDII) disorders, six studies included only BDI and one study only BDII participants. Substance abuse was an exclusion criterion in nine studies. Most studies did not exclude comorbidities, other than substance abuse. Consequently, the sample is a broad, ecologically valid, and generalizable representation of BD. All participating sites received approval from local ethics committees, and all participants provided written informed consent.

### Data acquisition and segmentation

High-resolution T1-weighted brain anatomical MRI scans were acquired at each site, see Table S5. All groups used the same analytical protocol and performed the same visual and statistical quality assessment, as listed at: http://enigma.ini.usc.edu/protocols/imaging-protocols/. These protocols are standardized across the consortium, are open-source, and available online for anyone to scrutinize, in order to foster open science/replication/reproducibility. They were applied in the previous publications by our group [5, 23] and more broadly in large-scale ENIGMA studies of major depression, schizophrenia, ADHD, OCD, PTSD, epilepsy, and autism [37].

Briefly, using the freely available and extensively validated FreeSurfer software, we performed segmentations of 8 subcortical regions (lateral ventricles, nucleus accumbens, amygdala, hippocampus, pallidum, putamen, caudate nucleus, and thalamus), per hemisphere (left and right), based on the Desikan–Killiany atlas. We also computed measures of total intracranial volume (ICV) to standardize estimates. Visual quality controls were performed on a region of interest (ROI) level aided by a visual inspection guide including pass/fail segmentation examples. In addition, we generated diagnostic histogram plots for each site and outliers, i.e., ROI volumes, which deviated from the site mean for each structure at >3 standard deviations, were flagged for further review. All ROIs failing quality inspection were withheld from subsequent analyses, see Table S6. Previous analyses from the ENIGMA-BD Working Group showed that scanner field strength, voxel volume, and the version of FreeSurfer used for segmentation did not significantly influence the effect size estimates. Further details regarding these analyses, as well as forest plots of subcortical effect sizes from individual sites, can be found here [23].

We focused on subcortical volumes, as these regions, including amygdala [20, 24, 38], hippocampus [19, 20, 38–40], striatum [19, 20, 39, 41], thalamus [19, 20, 24, 39, 40] or lateral ventricles [42] were previously associated with obesity. The same subcortical regions are consistently associated with BD in meta-analyses [9, 43] and mega-analyses [23, 44]. In addition, larger lateral ventricles are among the most replicated findings in BD [7, 44]. Analyses of other structural measures, i.e., cortical thickness, surface area, subcortical shape, were beyond the scope of the current study and will be analyzed separately.

### Statistical modeling

We used linear mixed modeling (package *nlme* version 3.1-140 in *R* version 3.6.2) with individual subcortical volumes as dependent variables and with (1) group (BD vs no BD), or (2) BMI and in each case also age, sex, hemisphere (left or right), and total ICV as fixed predictors. Models also included random effects of hemisphere within participants and a random effect of the data collection site. This random effect structure captures inter-subject variability, inter-hemisphere volume differences within subjects, and variability across data collection sites. Improvements in the Akaike information criterion supported this approach. We created one model per each of the eight subcortical regions, each including both hemispheres and all of the covariates, as described above. Subsequently, we modeled both group and BMI jointly, alongside the above-described covariates. We tested for interactions and included them where significant. In order to compare the associations with brain measures across the sites, we also separately tested for BMI × site interaction. We used BMI as a continuous variable, which captures more variability between participants, increases sensitivity, and was the preferred approach in most previous studies [21]. BMI was normally distributed, see Fig. S1. We checked the normality of model residuals using QQ plots and tested for multicollinearity using the variance inflation factor (VIF) of all predictor variables included in the modeling, see Table S7. Variance in regional volumes was comparable between groups, differences ranging between 2 and 15%.

In post hoc analyses among individuals with BD, we separately explored the effects of medications. As the rates of monotherapy were low in this sample, we studied the association between number of medication classes used (zero through three, including anticonvulsants, antipsychotics, and antidepressants) and BMI or subcortical volumes. We also separately modeled the effects of current lithium (Li) treatment. We used the same covariates and random effect structure as described above. The a priori decision to analyze the effects of Li separately was motivated by the fact that Li predominantly shows a positive association with subcortical volumes [8, 45], whereas antipsychotics [46, 47] or anticonvulsants [48] are predominantly negatively associated with regional brain volumes. In our previous work, we have documented that analyzing Li-treated individuals together with those not on Li may cancel the volumetric alterations and nullify effect sizes [8].

We adjusted all *p* values for multiple comparisons using false discovery rate (FDR), with adjusted *p* values reported, at *α* = 0.05. We calculated effect sizes for between-group differences (Cohen’s *d*) and associations between BMI and ROI volumes (partial *r*), together with their 95% confidence intervals (CIs) using model coefficients and their standard error (SE) [49], as also used in previous ENIGMA studies. The computer code for all of these analyses will be provided upon reasonable request.

### Mediation analysis

We tested whether the variance in ventricular volume associated with a diagnostic group (direct path) remained significant after also accounting for variance associated with BMI (indirect path). The presence or absence of BD was modeled as the associated variable, BMI as the mediating variable, and regional brain volume was the dependent variable. We modeled the direct effect of group on volume, in comparison with the indirect effect of this association through BMI as a mediator, corrected for age, sex, ICV, and random effects. To test this, we built 5000 bootstrapped models using random selection with replacement. This method nonparametrically identified the 95% CI for effect sizes. The bootstrap CI, which did not include zero indicated a significant indirect effect. Simulation research indicates that the bootstrap method is more robust to non-normality and has better type I error control than the Sobel test [50]. Nevertheless, for methodological consistency, we also applied the Sobel test to investigate whether accounting for BMI significantly mitigated group-related differences in volume. All of these analyses were performed in *R* (version 3.6.2).

These analyses were applied only to regions, which met the criteria for mediation, i.e., showed that: (1) BD was a significant predictor of the ROI volume, (2) BD was a significant predictor of the mediator (BMI), and when modeled jointly, (3) the mediator was a significant predictor of the dependent variable, and (4) the strength of the coefficient of the previously significant independent variable (BD) was reduced.

## Results

### Sample description

We included 2735 participants (1134 individuals with BD and 1601 healthy controls), see Table 1.

**Table 1:** Demographic, diagnostic and treatment characteristics of the sample.

	Controls	Cases	Significance
*N*	1601	1134	
Age, mean (SD)	35.47 (12.63)	41.72 (12.66)	*t*(2 436) = 12.73, *p* < 0.001
BMI, mean (SD)	24.43 (4.12)	26.80 (5.22)	*t*(2070)^a^ = 12.71, *p* < 0.001
Normal weight/ Overweight/ Obese, *N* (%)	1014 (63.34)/ 437(27.30)/ 150 (9.37)	470 (41.45)/ 399(35.19)/ 265 (23.37)	*χ*2 = 157.87, DF = 2, *p* < 0.001
Sex, *N* (%) female	916 (57.21)	684 (60.32)	*χ*2 = 2.63, *p* = 0.105
Diagnosis, *N* (%)			N/A
BDI	-	777 (68.52)	
BDII	-	258 (22.75)	
BD-NOS	-	3 (0.26)	
Treatment at time of scanning, *N* (%)/ Monotherapy N(%)			N/A
No treatment	-	79 (6.97)	
Lithium	-	516 (45.5)/112 (9.88)	
Antiepileptic	-	382 (33.69)/51 (4.50)	
First-generation antipsychotic	-	68 (6)/5 (0.44)	
Second-generation antipsychotic	-	349 (30.78)/39 (3.44)	
Antidepressant	-	380 (33.51)/28 (2.47)	
Mood state, *N* (%)			N/A
Euthymic	-	595 (52.47)	
Depressed	-	287 (25.31)	
Manic	-	28 (2.47)	
Hypomanic	-	10 (0.88)	
Mixed	-	5 (0.44)	
Age of onset, mean (SD)	-	25.16 (10.83)	N/A
History of psychosis, *N* (%)	-	423 (37.3)	N/A

^a^There were no missing age or BMI values. We used the Welch two-sample *t*-test (unequal variance assumed), which relies on a Welch–Satterthwaite degrees of freedom adjustment, resulting in varying degrees of freedom.

### Regional volume differences by group

**Fig. 1:**
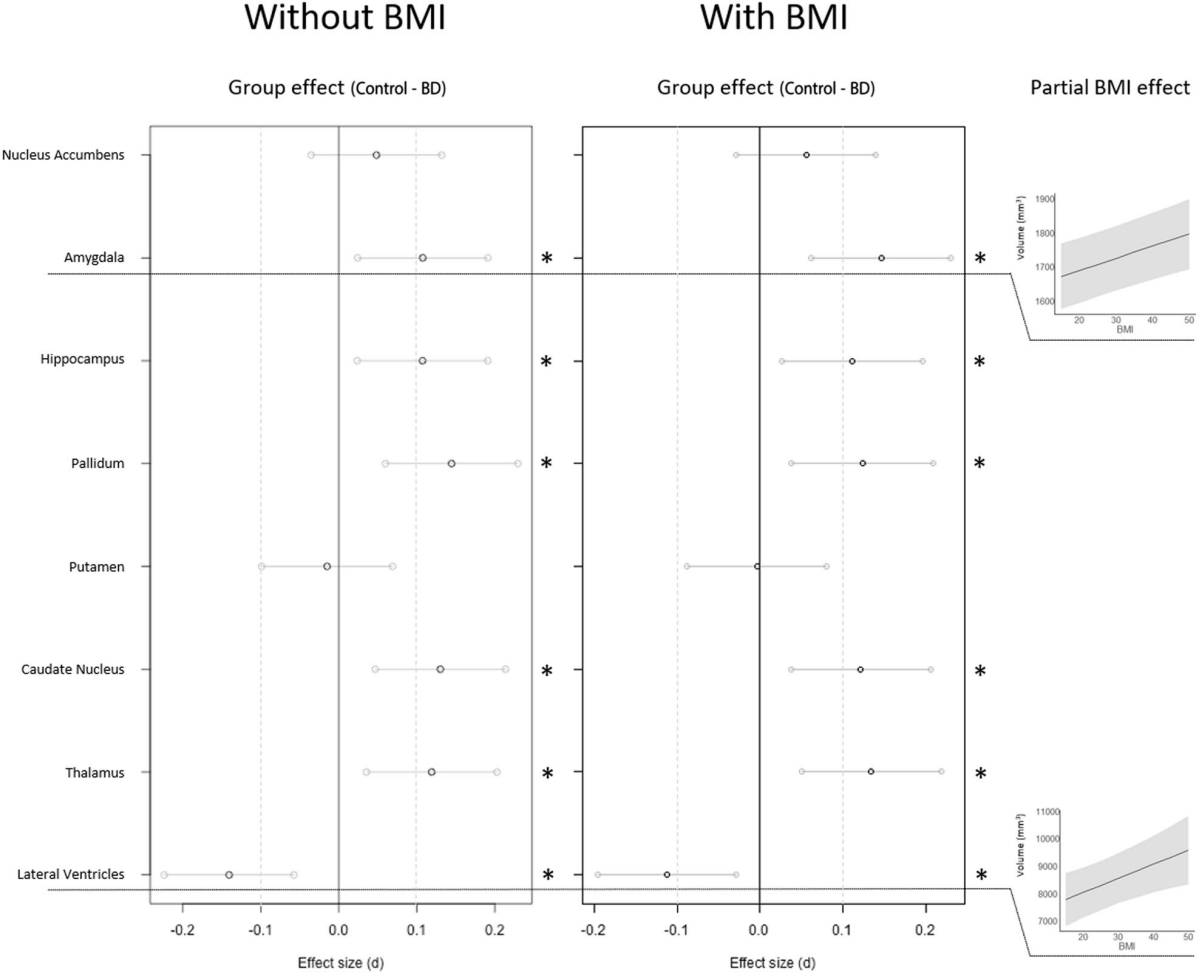
Effect size of between-group volume differences in each region without adjusting for BMI (left), and after adjusting for BMI (right). Statistically  significant group differences are denoted by asterisks. BMI slopes shown where significant (FDR-adjusted *p* < 0.05).

BMI, when modeled without the diagnosis factor, was associated positively with the volume of the lateral ventricles and amygdala, and negatively with pallidal volume, see Table 2. The association between BMI and these subcortical measures was linear, see Fig. S2. The diagnosis of BD, when modeled without BMI, was associated with larger lateral ventricular volumes, and smaller volumes of the amygdala, hippocampus, pallidum, caudate nucleus, and thalamus, see Fig. 1 and Table 2.

**Table 2:** Results of the multiple regression analyses.

Region	*b*	SE *b*	DF	*p* (FDR)		*d*	95% CI		*b*	SE b	DF	*p* (FDR)		*r*	95% CI	
	Effect of diagnosis without BMI								Effect of BMI, without diagnosis							
Accumbens	4.66	4.09	2372	0.290		0.05	−0.04	0.13	0.23	0.39	2372	0.630		0.01	−0.03	0.05
Amygdala	21.70	8.62	2380	0.016	*	0.11	0.02	0.19	2.96	0.81	2380	0.002	*	0.08	0.04	0.12
Hippocampus	44.49	17.68	2386	0.016	*	0.11	0.02	0.19	0.03	1.66	2386	0.983		0.00	−0.04	0.04
Pallidum	34.21	10.35	2274	0.004	*	0.14	0.06	0.23	−2.60	0.97	2274	0.020	*	−0.06	−0.10	−0.02
Putamen	−9.52	24.89	2351	0.702		−0.02	−0.10	0.07	3.48	2.36	2351	0.226		0.03	−0.01	0.07
Caudate	57.37	18.61	2388	0.005	*	0.13	0.05	0.22	−2.75	1.75	2388	0.226		−0.03	−0.07	0.01
Thalamus	82.77	29.78	2382	0.010	*	0.12	0.04	0.20	3.49	2.80	2382	0.283		0.03	−0.02	0.07
Lat. Ventricles	−613.65	187.28	2414	0.004	*	−0.14	−0.22	−0.06	61.22	17.61	2414	0.002	*	0.07	0.03	0.11
	Partial effect of diagnosis, when controlling for BMI								Partial effect of BMI, when controlling for diagnosis							
Accumbens	5.38	4.17	2371	0.225		0.06	−0.03	0.14	0.33	0.40	2371	0.455		0.02	−0.02	0.06
Amygdala	29.70	8.78	2379	0.008	*	0.15	0.06	0.23	3.55	0.82	2379	0.000	*	0.09	0.05	0.13
Hippocampus	46.59	18.08	2385	0.013	*	0.11	0.03	0.20	0.95	1.69	2385	0.577		0.01	−0.03	0.05
Pallidum	29.76	10.58	2273	0.010	*	0.12	0.04	0.21	−2.02	0.99	2273	0.112		−0.04	−0.08	0.00
Putamen	−2.26	25.40	2350	0.929		0.00	−0.09	0.08	3.43	2.41	2350	0.248		0.03	−0.01	0.07
Caudate	53.62	19.02	2387	0.010	*	0.12	0.04	0.21	−1.71	1.78	2387	0.449		−0.02	−0.06	0.02
Thalamus	94.66	30.44	2381	0.008	*	0.13	0.05	0.22	5.35	2.86	2381	0.122		0.04	0.00	0.08
Lat. Ventricles	−500.84	191.11	2413	0.013	*	−0.11	−0.20	−0.03	51.54	17.98	2413	0.016	*	0.06	0.02	0.10

*p < 0.05

**Fig. 2:**
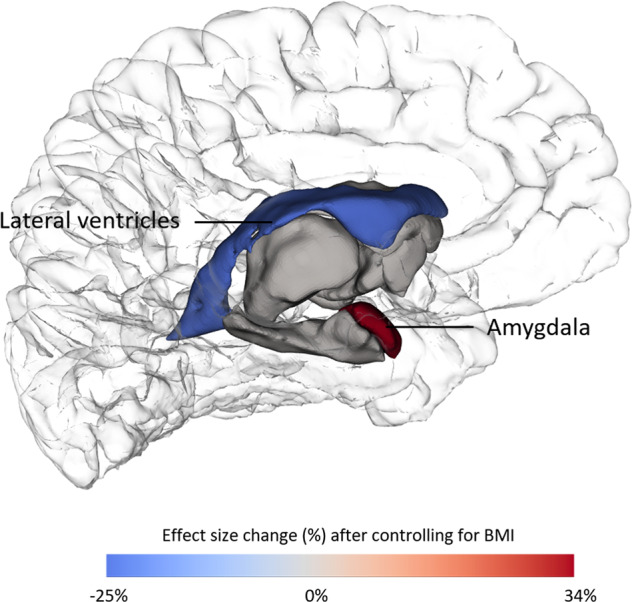
Changes in differences between BD and control individuals with versus without controlling for BMI. Change in group effect size after controlling for BMI, shown in regions where both BD and BMI were significantly associated with regional volume.

The impact of jointly modeling BMI and BD varied by region, see Fig. 1 and Table 2. In the lateral ventricles and amygdala, both BD and BMI remained significantly associated with regional volumes when analyzed jointly. Adjusting for BMI decreased the BD vs control differences in ventricular volume, but it increased the group differences in amygdala volumes, see Table 2 and Fig. 2. In the pallidum, the partial effect of BMI when adjusting for BD became non-significant. In all other regions, BD remained significantly associated with regional brain volume even while controlling for BMI. There was no significant interaction between group and BMI, or between BMI and site, see Table S8 and  Fig. S3.

### Mediating effect of BMI

**Fig. 3:**
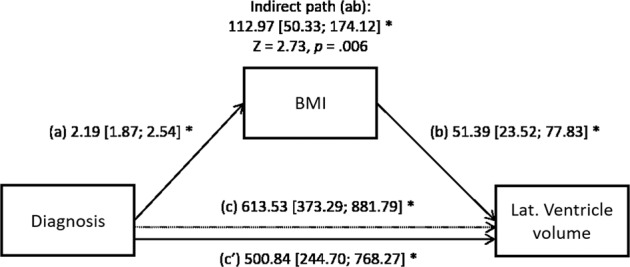
The effect of diagnosis and BMI on ventricular volume. Path (c) represents the direct effect of diagnosis, while (a) through (b) represents the indirect path of diagnosis through BMI. The adjusted effect of diagnosis on volume is shown after accounting for BMI (c′). We show unstandardized coefficients along with their 95% CI derived from bootstrap. Significant effects (*p* < 0.05) are marked by asterisks. In all effects, we controlled for the covariates age, sex, and data collection site, while those impacting volume additionally adjusted for hemisphere, ICV.

Only the lateral ventricles met conditions for mediation analyses, i.e., BD was associated with both BMI and ventricular volume, but the partial effect of BD on ventricular volume decreased when the significant partial effect of BMI was included in the model (Fig. 3). There was a significant indirect effect of BD associated with larger ventricle volumes through BMI (112.97; 95% CI, 50.33-174.12, see Fig. 3). Specifically, 18.41% (95% CI: 5.71; 46.64) of the association between diagnosis and ventricular volume was mediated by BMI (*Z* = 2.73, *p* = 0.006, Fig. 3).

### Medications, clinical variables, BMI, and brain structure

In individuals with BD, higher BMI was associated with a higher number of medication classes per participant (*t*(1100) = 4.89, *p* < 0.001), but not with Li treatment (t(1030) = −0.42, *p* = 0.67). The number of medication classes was also significantly associated with lateral ventricular volume (*b* = 485.89, SE *b* = 160.46, *t*(1099) = 3.03, *p* = 0.003), but no other regional volumes. Jointly modeling the association between number of medications, BMI and ventricular volumes yielded a significant partial effect of number of medications (*b* = 459.76, SE *b* = 162.18, *t*(1098) = 2.83, *p* = 0.005), whereas the partial effect of BMI was non-significant (*b* = 29.81, SE *b* = 26.16, *t*(1098) = 1.14, *p* = 0.255). There was no interaction between BMI and medications (*t*(1097) = 0.908, *p* = 0.364). The model that included both BMI and medications achieved a fit (*R*^2^ = 22.76%) very similar to the model which included only BMI (*R*^2^ = 22.40%) or only the number of medications (*R*^2^ = 22.69%). Thus, combining the two factors offered minimal unique value to the model, despite very low multicollinearity (BMI VIF = 1.01, medications VIF = 1.01). BMI was not significantly associated with illness duration, history of psychotic symptoms, diagnostic subtype, or mood state; see Table S9.

## Discussion

In this study of 2735 individuals, BMI was associated with similar regional brain volumes as BD, including lateral ventricles, amygdala, and pallidum. Those with higher BMI demonstrated larger volumes of ventricles or amygdala than those with lower BMI, despite having the same diagnosis of BD. About one-fifth of the total association between BD and ventricular volume was related to the higher BMI in BD. Other subcortical regions, including hippocampus, caudate, and thalamus, were robustly associated with BD even when we controlled for BMI. Importantly, this large study showed no interaction between BD and BMI in their relationship to subcortical brain volumes, indicating that the effects of BMI on subcortical volumes were comparable between BD and control individuals. Last but not least, increased BMI and the number of psychiatric medications mostly overlapped in their contribution to larger ventricular volumes in BD.

The unique focus of this study was to investigate how apparent regional brain volume differences between individuals with and without BD may change when adjusting for BMI. Controlling for BMI decreased apparent neurostructural differences in ventricular volumes, that had been attributed to the diagnosis of BD. In fact, a significant proportion of the total association between BD and ventricular volume was related to higher BMI. This is the first study to suggest that higher BMI may in part account for larger ventricles, one of the most replicated findings in BD [7, 19, 33]. While surprising, this is in keeping with a study in major depressive disorders, which also showed that BMI contributed to volumetric alterations in depression [51]. Differences in ventricular volumes between individuals with BD and controls may in fact be smaller and less consistent than previously reported and may in part depend on factors other than the diagnosis of BD.

It is reassuring that previously reported associations between BD and smaller volumes of hippocampus, caudate, and thalamus were robust to controlling for BMI. Furthermore, our study demonstrated that adjusting for BMI improved sensitivity to between-group differences in amygdala volumes. Interestingly, previous meta-analyses of amygdala volumes demonstrated an absence of between-group differences in a number of individual studies as well as a significant statistical heterogeneity [9, 23]. This is congruent with the presence of a hidden variable, such as BMI. Thus, not controlling for BMI could have contributed to false-negative findings in previous studies. Differences in amygdala volumes between individuals with BD and controls may be potentially larger and more consistent than previously reported.

This is also one of the first studies to investigate the interplay between medications, BMI, and brain structure. As in other studies, we showed that antipsychotics and anticonvulsants were negatively associated with brain structure [23, 35, 46–48, 52, 53] and positively with BMI [33, 34]. Thus, some authors have proposed that the association between medications, especially antipsychotics and brain structure could be confounded by weight gain [54]. A single previous study showed that antipsychotic medications remained associated with brain structure even when BMI was controlled for [55], which is in keeping with our findings. Here we also demonstrated that when modeled separately, each of BMI and the number of medications explained a similar and largely overlapping proportion of variance in ventricular volumes. Thus, we cannot rule out that weight gain may be relevant for some of the negative effects of medications on the brain structure. This needs to be verified in a prospective study. Preclinical studies should also investigate whether some of the mechanisms through which antipsychotics contribute to obesity, i.e. upregulation of neuropeptide Y and melanin-concentrating hormone, decreased expression of leptin-induced AMP-activated protein kinase, reduction of orexin, effects on α2 or muscarinic receptors [56], could also directly affect brain structure.

Our findings are consistent with previous large-scale studies, which also demonstrated negative associations between BMI and the volume of subcortical regions including pallidum [19, 20, 39, 57], and the temporal lobes overall [28, 29], but a positive association with amygdala volume [19, 24, 39, 57]. Yet, we do not know the temporal direction or pathophysiology of these findings. It is possible that overweight/obesity caused the observed changes through a range of mechanisms, including effects of adipokines [58], oxidative stress, systemic inflammation [59, 60], insulin resistance/diabetes [16, 27], hypertension [15, 39] or dyslipidemia [60]. Smaller brain volumes in obesity may also reflect lower mobility/fitness or sedentary lifestyle, which are associated with the volumes of hippocampus [61] or motoric brain regions, including striatum [62–64]. However, the reverse causality, where neurostructural alterations cause obesity, is also possible. Specific brain changes may increase the risk of obesity through impulsivity, conditioning, or impaired homeostatic regulation [65], and these same brain alterations may be overrepresented in BD [22].

We can find some insight into these questions from the neuroanatomical patterns of the observed changes. The positive association between BMI and amygdala volume may support the role of neurostructural alterations in influencing obesity. The amygdala is involved in cue-triggered learning and Pavlovian conditioning to hedonic food that represents a key mechanism in future weight gain [66]. Indeed, previous studies have shown that obese individuals exhibit hyperactive responses to food cues in several regions, including the amygdala [67, 68] and that this response correlated with BMI [67, 69]. The amygdala is also implicated in appetitive behavior in preclinical studies [70, 71]. The positive association between BMI and ventricular volume, which summarizes atrophy across surrounding subcortical regions, may indicate a more non-specific, diffuse effect, which might be congruent with brain alterations as consequences of higher BMI. The negative effects of BMI on brain structure are supported by replicated evidence from different lines of investigation, including a Mendelian randomization study [72], several longitudinal studies, including one in BD, which have demonstrated that obesity or obesity-related metabolic alterations precede and accelerate brain changes over time, including temporal lobe atrophy and expansion of lateral ventricles [30, 73, 74]. Regardless of the exact mechanisms and temporal direction of the association, the findings have important implications.

Considering the high prevalence of obesity, which has reached epidemic proportions, especially in major psychiatric disorders, studying the links between obesity, BD, and brain structure could have major clinical implications. If obesity leads to brain alterations, this represents a manageable/modifiable risk factor for neuroprogressive BD [75]. Obesity-related structural brain abnormalities might be preventable or even reversible with dietary/lifestyle/surgical interventions focused on weight management [76–78]. Also, medications targeting obesity, such as liraglutide, may have neuroprotective effects, as also documented in participants with BD [79]. The links between obesity and brain structure might provide new treatment options for some of the currently difficult to treat outcomes, such as cognitive impairments, residual symptoms, poor functioning, which have also been associated with obesity [1, 80] or neurostructural alterations/ventriculomegaly [81, 82]. On the other hand, if certain BD-related alterations predispose individuals to obesity, then this is a prognostic marker for targeted prevention of obesity in BD. Indeed, previous studies have documented that it is possible to differentiate obese from normal weight subjects based on multivariate brain structural patterns [24].

These findings could also help explain the heterogeneity of brain imaging findings in BD. The extent of brain imaging alterations in several relevant regions was contingent not only on the presence of BD but also on the variations in an additional factor, i.e., BMI. Thus, BD individuals with higher BMI will show larger ventricles or amygdala then BD individuals with lower BMI. Similarly, differences between BD and control individuals in ventricular or amygdala volumes will in part depend on between-group differences in BMI. Consequently, variations in BMI could help explain why brain imaging measures vary within the same diagnosis [5] and why the magnitude of patient-control differences varies across studies in BD [6–10].

Our results could also provide insight into the marked overlap among major psychiatric disorders in brain imaging alterations [83, 84]. For example, greater rates of obesity [33] and larger ventricles [85] are also reported in schizophrenia. It is possible that some of the overlaps among major psychiatric disorders in their brain imaging findings are related to overlaps in comorbid medical conditions, including obesity. These common influences could even be obscuring effects that are truly disorder specific.

With 2735 individuals, this is the largest study investigating associations between BD, BMI, and brain structure and the largest mega-analysis of subcortical volumes in BD. Of note, the previous ENIGMA meta-analysis [23] failed to detect significantly smaller pallidum and caudate in BD, likely due to lower statistical power relative to our mega-analysis. Our focus on BMI as a specific mediator of neurostuctural alterations in those suffering from BD or exposed to psychiatric medications targeted important knowledge gaps. In addition to novel findings, we provide several replications of previous results, including similar associations between subcortical brain structure and BMI or BD. Due to the large sample size and multicentric, international nature of the study, these results may be considered highly generalizable, as the included individuals represent a broad spectrum of BD from around the world.

This study has several limitations. Due to the focus on legacy datasets, we could not analyze specific anthropometric or metabolic markers beyond BMI. Waist circumference or waist–hip ratio may show more extensive associations with GM, but usually in the same regions [20, 86]. Moreover, BMI is much easier to acquire, it captures a large part of variance in other obesity-related alterations and is by far the most frequently used measure [16, 21], thus allowing for a more direct comparison with previous work. Furthermore, in our previous study, insulin resistance or diabetes were not associated with amygdala or pallidum volume [27]. As the study was performed in 13 countries, it is possible that racial/ethnic and social-economic factors could have contributed to our findings, but there was no interaction between BMI and site in their effects on brain measures. Due to the nature of ENIGMA, which works with legacy datasets, we could not access raw, whole-brain data and could not utilize methods, such as voxel-based morphometry. We did not focus on subregions, which are often beyond the resolution of MRI or cannot be reliably delineated without dedicated and often very lengthy scans. Aside from the standardization of methods, we also addressed any differences between scanners statistically by using mixed models and including site as a random factor in all analyses. As our study was not designed to test the effects of medication, the number of medication classes prescribed could also be a proxy for the severity/complexity of illness. Medication details were limited to the current prescription, without any measures of duration, dosage, compliance, treatment response, or symptom levels at the time of prescription, so we cannot address the effects of these factors. Fat content near the MRI coil may lead to slight signal intensity changes [87], but the vast majority of individuals were normal weight to overweight. Last but not least, caution is needed when interpreting mediation analyses in observational studies.

## Conclusions

To conclude, we confirmed regionally specific associations between BMI and subcortical volumes in individuals with BD. Variations in BMI contributed to variations in regional brain volumes, which in case of ventricles increased, but in case of amygdala decreased apparent differences between BD and control individuals. Higher BMI may even in part account for larger ventricles, one of the most replicated findings in BD. Volumes of hippocampus, caudate and thalamus remained smaller in BD regardless of BMI. Our findings, together with the high rates of obesity in BD indicate that measures of obesity should be incorporated in future neuroimaging investigations of BD in order to decrease their heterogeneity. The fact that a significant proportion of the association between BD and ventricular volume was related to higher BMI raises the possibility that targeting BMI could lower the extent of ventricular expansion in BD. Future studies should prospectively investigate whether obesity could be a modifiable risk factor for neuroprogression and related adverse clinical outcomes.

## Supplementary information

Supplemental figures and analyses
