# Supplementary material for: Association between body mass index and subcortical brain volumes in bipolar disorders–ENIGMA study in 2735 individuals
Source: Mol Psychiatry. 2021 Apr 16;26(11):6806–19. doi: 10.1038/s41380-021-01098-x (PMC8760047; doi:10.1038/s41380-021-01098-x)
Supplement: Supplementary file 1 — Supplemental figures and analyses [file 41380_2021_1098_MOESM1_ESM.docx]

**Table S1:** Descriptive statistics of included samples. Abbreviations: BD (bipolar disorder), Li (lithium), AED (antiepileptics), FGA (first generation antipsychotics), SGA (second generation antipsychotics), AD (antidepressants).

|  | **NUIG** | | **Marburg** | | **Barcelona** | | **UMCU** | | **Muenster** | | **MNC** | |
| --- | --- | --- | --- | --- | --- | --- | --- | --- | --- | --- | --- | --- |
|  | Control | Case | Control | Case | Control | Case | Control | Case | Control | Case | Control | Case |
| N | 57 | 55 | 355 | 47 | 19 | 49 | 90 | 119 | 216 | 25 | 384 | 39 |
| Age, mean (SD) | 41.89 (9.76) | 42.18 (9.78) | 35.34 (12.76) | 43.7 (10.87) | 42.53 (7.56) | 43.12 (7.65) | 46.02 (13.36) | 46.27 (12.71) | 28.37 (10.36) | 41.68 (13.23) | 39.09 (11.28) | 39.59 (12.5) |
| Sex, N (%) female | 28 (49.12) | 28 (50.91) | 225 (63.38) | 31 (65.96) | 13 (68.42) | 28 (57.14) | 43 (47.78) | 56 (47.06) | 141 (65.28) | 7 (28) | 197 (51.3) | 18 (46.15) |
| *Diagnosis, N (%)* |  |  |  |  |  |  |  |  |  |  |  |  |
| BD-I | - | 55 (100) | - | 32 (68.09) | - | 49 (100) | - | 119 (100) | - | 8 (32) | - | 28 (71.79) |
| BD-II | - | 0 (0) | - | 15 (31.91) | - | 0 (0) | - | 0 (0) | - | 17 (68) | - | 11 (28.21) |
| BD-NOS | - | 0 (0) | - | 0 (0) | - | 0 (0) | - | 0 (0) | - | 0 (0) | - | 0 (0) |
| *Treatment, N (%)* |  |  |  |  |  |  |  |  |  |  |  |  |
| Li | - | 42 (76.36) | - | 10 (21.28) | - | 35 (71.43) | - | 81 (68.07) | - | 7 (28) | - | 15 (38.46) |
| AED | - | 15 (27.27) | - | 17 (36.17) | - | 26 (53.06) | - | 19 (15.97) | - | 6 (24) | - | 12 (30.77) |
| FGA | - | 2 (3.64) | - | 1 (2.13) | - | 2 (4.08) | - | 3 (2.52) | - | 1 (4) | - | 2 (5.13) |
| SGA | - | 4 (7.27) | - | 18 (38.3) | - | 26 (53.06) | - | 46 (38.66) | - | 14 (56) | - | 27 (69.23) |
| AD | - | 11 (20) | - | 17 (36.17) | - | 9 (18.37) | - | 28 (23.53) | - | 13 (52) | - | 39 (100) |
| *Mood state, N (%)* |  |  |  |  |  |  |  |  |  |  |  |  |
| Euthymic | - | 50 (90.91) | - | 12 (25.53) | - | 49 (100) | - | - | - | 5 (20) | - | 0 (0) |
| Depressed | - | 5 (9.09) | - | 21 (44.68) | - | 0 (0) | - | - | - | 11 (44) | - | 39 (100) |
| Manic | - | 0 (0) | - | 8 (17.02) | - | 0 (0) | - | - | - | 2 (8) | - | 0 (0) |
| Hypomanic | - | 0 (0) | - | 2 (4.26) | - | 0 (0) | - | - | - | 6 (24) | - | 0 (0) |
| Mixed | - | 0 (0) | - | 0 (0) | - | 0 (0) | - | - | - | 1 (4) | - | 0 (0) |
| Onset, mean (SD) | - | 28.27 (8.33) | - | 23.98 (11.33) | - | 25.8 (8.43) | - | 29.74 (10.63) | - | 25.84 (12.4) | - | 27.31 (9.16) |
| Psychosis, N (%) | - | 51 (92.73) | - | - | - | 38 (77.55) | - | 90 (75.63) | - | - | - | - |
|  | **Tulsa** | | **Sydney** | | **Malt** | | **CHRM2** | | **CIAM** | | **Halifax** | |
|  | Control | Case | Control | Case | Control | Case | Control | Case | Control | Case | Control | Case |
| N | 91 | 70 | 78 | 53 | 44 | 44 | 47 | 37 | 31 | 25 | 62 | 98 |
| Age, mean (SD) | 32.37 (10.08) | 38.26 (11.68) | 22.41 (3.86) | 25.04 (3.61) | 31.18 (9.12) | 34.36 (7.31) | 38.66 (13.11) | 42.22 (12.38) | 26.58 (4.87) | 29.64 (5.19) | 37.46 (12.67) | 48.49 (14.58) |
| Sex, N (%) female | 56 (61.54) | 56 (80) | 40 (51.28) | 37 (69.81) | 26 (59.09) | 32 (72.73) | 27 (57.45) | 18 (48.65) | 14 (45.16) | 10 (40) | 40 (64.52) | 56 (57.14) |
| *Diagnosis, N (%)* |  |  |  |  |  |  |  |  |  |  |  |  |
| BD-I | - | 34 (48.57) | - | 31 (58.49) | - | 0 (0) | - | 29 (78.38) | - | 25 (100) | - | 67 (68.37) |
| BD-II | - | 31 (44.29) | - | 22 (41.51) | - | 44 (100) | - | 8 (21.62) | - | 0 (0) | - | 31 (31.63) |
| BD-NOS | - | 5 (7.14) | - | 0 (0) | - | 0 (0) | - | 0 (0) | - | 0 (0) | - | 0 (0) |
| *Treatment, N (%)* |  |  |  |  |  |  |  |  |  |  |  |  |
| Li | - | - | - | 15 (28.3) | - | 3 (6.82) | - | 11 (29.73) | - | 12 (48) | - | 54 (55.1) |
| AED | - | - | - | 30 (56.6) | - | 22 (50) | - | 1 (2.7) | - | 15 (60) | - | 19 (19.39) |
| FGA | - | - | - | 0 (0) | - | 2 (4.55) | - | 1 (2.7) | - | 8 (32) | - | 0 (0) |
| SGA | - | - | - | 16 (30.19) | - | 4 (9.09) | - | 23 (62.16) | - | 10 (40) | - | 16 (16.33) |
| AD | - | - | - | 25 (47.17) | - | 15 (34.09) | - | 15 (40.54) | - | 2 (8) | - | 19 (19.39) |
| *Mood state, N (%)* |  |  |  |  |  |  |  |  |  |  |  |  |
| Euthymic | - | - | - | 50 (94.34) | - | 24 (54.55) | - | 37 (100) | - | 25 (100) | - | 82 (83.67) |
| Depressed | - | - | - | 2 (3.77) | - | 19 (43.18) | - | 0 (0) | - | 0 (0) | - | 0 (0) |
| Manic | - | - | - | 0 (0) | - | 0 (0) | - | 0 (0) | - | 0 (0) | - | 0 (0) |
| Hypomanic | - | - | - | 0 (0) | - | 1 (2.27) | - | 0 (0) | - | 0 (0) | - | 0 (0) |
| Mixed | - | - | - | 1 (1.89) | - | 0 (0) | - | 0 (0) | - | 0 (0) | - | 0 (0) |
| Onset, mean (SD) | - | - | - | 15.06 (3.36) | - | 15.98 (5.8) | - | 26.48 (10.16) | - | 21.54 (4.37) | - | 21.54 (6.67) |
| Psychosis, N (%) | - | - | - | 16 (30.19) | - | - | - | 4 (10.81) | - | 25 (100) | - | 18 (18.37) |
|  | **Haarman** | | **SBP** | | **Milano** | | **Medellin** | | **SCDS** | |  |  |
|  | Control | Case | Control | Case | Control | Case | Control | Case | Control | Case |  |  |
| N | 0 | 22 | 83 | 130 | 0 | 200 | 0 | 75 | 44 | 46 |  |  |
| Age, mean (SD) | - | 44.5 (10.52) | 39.13 (14.57) | 40.42 (12.24) | - | 46.76 (11.24) | - | 40.19 (11.78) | 34.07 (10.86) | 33.98 (10.75) |  |  |
| Sex, N (%) female | - | 12 (54.55) | 43 (51.81) | 80 (61.54) | - | 140 (70) | - | 48 (64) | 23 (52.27) | 27 (58.7) |  |  |
| *Diagnosis, N (%)* |  |  |  |  |  |  |  |  |  |  |  |  |
| BD-I | - | 22 (100) | - | 75 (57.69) | - | 149 (74.5) | - | 68 (90.67) | - | 46 (100) |  |  |
| BD-II | - | 0 (0) | - | 55 (42.31) | - | 48 (24) | - | 7 (9.33) | - | 0 (0) |  |  |
| BD-NOS | - | 0 (0) | - | 0 (0) | - | 3 (1.5) | - | 0 (0) | - | 0 (0) |  |  |
| *Treatment, N (%)* |  |  |  |  |  |  |  |  |  |  |  |  |
| Li | - | 13 (59.09) | - | 71 (54.62) | - | 101 (50.5) | - | 29 (38.67) | - | 17 (36.96) |  |  |
| AED | - | 12 (54.55) | - | 39 (30) | - | 80 (40) | - | 45 (60) | - | 24 (52.17) |  |  |
| FGA | - | 0 (0) | - | 5 (3.85) | - | 20 (10) | - | 11 (14.67) | - | 10 (21.74) |  |  |
| SGA | - | 6 (27.27) | - | 29 (22.31) | - | 45 (22.5) | - | 35 (46.67) | - | 30 (65.22) |  |  |
| AD | - | 6 (27.27) | - | 50 (38.46) | - | 113 (56.5) | - | 12 (16) | - | 6 (13.04) |  |  |
| *Mood state, N (%)* |  |  |  |  |  |  |  |  |  |  |  |  |
| Euthymic | - | 22 (100) | - | 130 (100) | - | 5 (2.5) | - | 58 (77.33) | - | 46 (100) |  |  |
| Depressed | - | 0 (0) | - | 0 (0) | - | 174 (87) | - | 16 (21.33) | - | 0 (0) |  |  |
| Manic | - | 0 (0) | - | 0 (0) | - | 18 (9) | - | 0 (0) | - | 0 (0) |  |  |
| Hypomanic | - | 0 (0) | - | 0 (0) | - | 0 (0) | - | 1 (1.33) | - | 0 (0) |  |  |
| Mixed | - | 0 (0) | - | 0 (0) | - | 3 (1.5) | - | 0 (0) | - | 0 (0) |  |  |
| Onset, mean (SD) | - | 20.27 (7.59) | - | 19.09 (11.32) | - | 31.1 (10.04) | - | 22.71 (9.95) | - | 29.67 (10.37) |  |  |
| Psychosis, N (%) | - | - | - | 63 (48.46) | - | 60 (30) | - | 58 (77.33) | - | - |  |  |

**Table S2:** Mean and standard deviation BMI for each group, and overall, at each data collection site. Inter-site differences in outcomes based on BMI or other factors were controlled in all models.

|  | BMI (Mean, SD) | | |
| --- | --- | --- | --- |
|  | BD | CN | Both |
| NUIG | 27.24 (4.95) | 25.59 (3.7) | 26.4 (4.42) |
| Marburg | 28.25 (6.5) | 24.27 (4.1) | 24.74 (4.62) |
| Barcelona | 27.22 (4.53) | 23.87 (3.32) | 26.28 (4.47) |
| UMCU | 26.56 (4.13) | 25.49 (3.68) | 26.1 (3.97) |
| Muenster | 26.01 (4.26) | 23.08 (3.62) | 23.38 (3.79) |
| MNC | 27.34 (5.41) | 24.63 (4.03) | 24.88 (4.24) |
| Tulsa | 28.57 (5.53) | 27.1 (5.08) | 27.74 (5.32) |
| Sydney | 26.8 (5.91) | 22.96 (3.37) | 24.52 (4.93) |
| Malt | 25.38 (4.33) | 23.38 (2.94) | 24.38 (3.81) |
| CHRM2 | 28.71 (7.13) | 25.53 (4.96) | 26.93 (6.18) |
| CIAM | 28.93 (5) | 23.2 (3.12) | 25.75 (4.95) |
| Halifax | 29.09 (5.54) | 26.15 (5.1) | 27.95 (5.54) |
| Haarman | 24.4 (3.36) | N/A | 24.4 (3.36) |
| SBP | 25.73 (4.63) | 24.1 (3.87) | 25.09 (4.41) |
| MilanoOSR | 25.4 (4.8) | N/A | 25.4 (4.8) |
| Medellin | 27.85 (5.14) | N/A | 27.85 (5.14) |
| SCDS | 24.66 (5.21) | 23.4 (3.43) | 24.05 (4.45) |

**Table S3:** Diagnostic instruments used to obtain diagnostic and clinical information

| Site | Diagnostic instruments used to obtain diagnostic and clinical information | Method for obtaining medication information |
| --- | --- | --- |
| Barcelona | Structured Clinical Interview for DSM-IV and Research Diagnostic Criteria (RDC). | Detailed clinical interview and review of case notes. |
| CHRM2 | Structured Clinical Interview for DSM-IV | Detailed clinical interview |
| CIAM | Structured Clinical Interview for DSM-IV for Axis I Diagnoses | Patient interview and Hospital records |
| Medellin (GIPSI) | Diagnostic Interview for Genetic Studies (DIGS) | Detailed clinical interview |
| Haarman (Moodinflame) | MINI 5.0.0 | Detailed clinical interview |
| Halifax | Structured Clinical Interview for DSM-IV for Axis I Diagnoses; (Halifax): Participants were recruited from patients followed up at a specialized Mood Disorders Program at Dalhousie University, Halifax, NS. The Program is a tertiary care clinic providing consultation services to family physicians and community psychiatrists and following up patients with BD. The diagnostic interviews were performed by pairs of clinicians, according to the Schedule for Affective Disorders and Schizophrenia, Lifetime version (SADS-L) and diagnoses were made according to DSM-IV criteria. | Questionnaire with self and interviewer reporting, in part using validated instruments; (Halifax): Patients had regular follow ups at the clinic, including monitoring of Li levels at least twice per year. Furthermore, we established illness course and treatment response to Li using NIMH life charts (NIMH-LCMTM) |
| Malt (Oslo) | Mini--International Neuropsychiatric Interview (MINI), DSM-IV criteria version 5.0. | Stanley Foundation Network Entry Questionnaire (NEQ). |
| FOR2107-Marburg and Muenster | Structured Clinical Interview for DSM-IV for Axis I Diagnoses | Self-report and hospital records |
| MNC | Structured Clinical Interview for DSM-IV for Axis I Diagnoses | Self-report and hospital records |
| MilanoOSR | Structured Clinical Interview for DSM-IV | Detailed clinical interview |
| NUIG | Structured Clinical Interview for DSMIV-TR-Patient Edition for patients and SCID_NP for controls | Detailed clinical interview outlining dose and duration of all psychotropic medication, supplemented by clinical notes where necessary. |
| SBP | Structured Clinical Interview for DSM-IV | Detailed clinical interview and review of clinical notes |
| SCDS (IMHBD) | Structured Clinical Interview for DSM-IV | Detailed clinical interview and review of case notes |
| Sydney | Diagnostic Interview for Genetic Studies (for 22-30 year-olds); Kiddie-SADS (for 12-21 year-olds) | Patient interview and Adult Health Screening questionnaire |
| Tulsa | Structured Clinical Interview for DSM-IV | Clinical interview |
| UMCU | Structured Clinical Interview for DSM-IV (SCID-I) for patients and Mini-International Neuropsychiatric Interview (M.I.N.I) for controls | Detailed clinical interview and/or diagnosis through treating physician |

**Table S4:** Exclusion criteria for study enrolment

| Site | Exclusion criteria for study enrolment |
| --- | --- |
| Barcelona | All patients with bipolar disorder were right handed. Exclusion criteria were age younger than 18 or older than 65 years, history of neurological disease or brain trauma, and alcohol/substance abuse in the 12 months prior to participation. Patients were also required to have a current IQ in the normal range (>70). All patients were diagnosed using DSM-IV and Research Diagnostic Criteria (RDC), based on a detailed clinical interview and review of case notes. All healthy controls met the same exclusion criteria as the patients, and they were interviewed and excluded if they reported a history of mental illness and/or treatment with psychotropic medication other than non-regular use of benzodiazepines or similar drugs for insomnia. They were also questioned about family history of mental illness and excluded if a first-degree relative had experienced symptoms consistent with major psychiatric disorder and/or had received any form of in- or outpatient psychiatric care. The healthy controls were selected to be matched with the patients on demographic variables and on premorbid IQ. |
| CHRM2 | CHRM2 Study: Inclusion criteria were 18-65 years of age. A diagnosis of BD (and Euthymia) was confirmed using the Diagnostic and Statistical Manual of Mental Disorders (DSM-IV-TR) Structured Clinical Interview for DSM Disorders (American Psychiatric Association, 1994) conducted by an experienced psychiatrist. Exclusion criteria included neurological disorders, learning disability, comorbid misuse of substance/alcohol and of axis-1 disorders, history of head injury resulting in loss of consciousness for >5 minutes along with a history of oral steroid use in the previous 3 months. Mood symptoms severity was assessed using the Hamilton Anxiety (HARS) and Depression (HDRS-21) Rating Scale and the Young Mania Rating Scale (YMRS) at MRI scanning. Healthy controls had no personal history of a psychiatric illness or history among first-degree relatives, defined using the Structured Clinical Interview for DSM-IV – Non-patient edition (American Psychiatric Association, 1994). |
| CIAM | Bipolar disorder participants were required to meet a diagnosis of bipolar I disorder with a significant history of psychosis. Between the ages of 19 and 40. Stable outpatients were recruited, and did not meet either mood polarity at the time of scanning. Were compatible for MRI and EEG imaging. Exclusion included history of epilepsy or seizures, which was an exclusion for EEG testing performed. Exclusion of any participants if presented with a significant/chronic general medication condition, e.g.. HIV, diabetes I/II, high blood pressure. Further for female participants no current/recent/suspected pregnancy or current lactation were allowed to participate. |
| Medellin (GIPSI) | Inclusion criteria: Diagnosis of Bipolar Disorder I or II based on DSM-IV-TR criteria, age between 18 and 60 years old, education level between 5 and 16 years. Exclusion criteria: Personal history of neurological disorders, mental disability, autism, electroconvulsive therapy and/or traumatic brain injury. |
| Haarman (Moodinflame) | In the MOODINFLAME study we included adult male and female subjects who were free of inflammation-related symptoms including fever and current or recent infectious or inflammatory disease, uncontrolled systemic disease, uncontrolled metabolic disease or other significant uncontrolled somatic disorders known to affect mood. They did not use somatic medication known to affect mood or the immune system, such as corticosteroids, non- steroid anti-inflammatory drugs and statins. Female candidates who were pregnant or recently gave birth were excluded. Patients and controls did not have a contraindication for MRI scanning. Patients were allowed to continue their regular psycho- pharmacological treatment. They were euthymic at the time of scanning as indicated by an Inventory of Depressive Symptoms - Clinician Version (IDS-C30) score o22 and a Young Mania Rating Scale (YMRS) score o12, respectively. Patients with any other current primary major psychiatric diagnosis were excluded including: schizophrenia, schizoaffective disorder, anxiety disorder and substance use disorders. HC did not have any current or life- time psychiatric diagnosis. |
| Halifax | Inclusion criteria. The BD patients (both Li and non-Li groups) had to have: (i) a diagnosis of bipolar I or II disorder made by a psychiatrist using the SCID; (ii) at least 10 years of illness; (iii) a history of at least five episodes of illness (including manic, depressive, or mixed episodes); (iv) current Hamilton Depression Rating Scale, 17-item version (HAM-D-17) score < 7; (v) current Young Mania Rating Scale (YMRS) score < 5; (vi) current Clinical Global Impressions Scale–Bipolar (CGI-BP) score < 3; and (vii) a period of euthymia for at least four months prior to scanning, as aside from state- related factors, patients in acute episodes may present with additional difficult to control confounding variables, including recent medication change or substance abuse. The non-Li group had to have less than three months of lifetime Li exposure, more than 24 months prior to the scanning. The Li group had to have a current Li treatment lasting a minimum of 24 months. Exclusion criteria. Individuals from any of the three groups were excluded if they met any of the magnetic resonance imaging (MRI) exclusion criteria or had any serious medical illness (e.g., brain injury, Cushings disease, or conditions treated with corticosteroids). Individuals with BD were excluded if they had: (i) more than one lifetime course of electroconvulsive therapy (ECT) or ECT in the previous 12 months; (ii) comorbid psychiatric disorders, and ⁄ or personality disorder; (iii) active substance abuse in the previous 12 months; (iv) significant change in their medication in the previous three months; or (v) current psychotic features or acute suicidality. Individuals from the non-Li group were excluded if they had: (i) Li exposure < 2 years before the scanning; or (ii) lifetime Li exposure of more than three months. The neuropsychiatrically healthy individuals were excluded if they had a personal history of psychiatric disorders. (Halifax) Diabetes Study: The subjects with BD were required to 1) have the diagnosis of bipolar I or II disorder made by a psychiatrist; and 2) be at least 18 years of age. Patients were excluded if they had 1) the diagnosis of organic mood disorder; 2) mood disorder not otherwise specified; or 3) more than one lifetime course of electroconvulsive therapy or electroconvulsive therapy within the last 6 months. The neuropsychiatrically healthy, euglycemic subjects were excluded if they had 1) a personal history of psychiatric disorders; or 2) T2DM. Subjects from any group were excluded if they 1) met any magnetic resonance imaging (MRI) exclusion criteria; 2) suffered from substance abuse in the last 12 months; had a history of 3) neurodegenerative disorders; or 4) cerebrovascular disease/stroke, as we were interested in the more subtle T2DM-related neuronal changes. Halifax High Risk Study: Families were identified through adult probands with BD, who had participated in 1) previous genetic and high-risk studies for the Halifax sample. Only the offspring from these families, not the probands, were a part of the MRI study. The offspring from BD parents were divided into two subgroups: 1) the Unaffected HR group, which consisted of 50 offspring with no lifetime history of psychiatric disorders. These individuals were at an increased risk for BD because they had one parent affected with a primary mood disorder. 2) The Affected Familial group, which consisted of 36 offspring who met criteria for a lifetime Axis I diagnosis of mood disorders (i.e., a personal history of at least one episode of depression, hypomania, or mania meeting full DSM-IV criteria). When available, we recruited more than one offspring per family. From this study, we provided data only from patients who had a personal history of bipolar disorder. |
| Malt (Oslo) | Inclusion criteria patients: A DSM-IV diagnosis of bipolar disorder type II. Exclusion criteria healthy controls: Controls with previous or current psychiatric illness were excluded from the study. The exclusion criteria for all participants were: A.) age younger than 18 or older than 50 years; B.) previous head injury with loss of consciousness for more than 1 minute; C.) history of neurological or other severe chronic somatic disorder; D.) pregnancy; E.) metallic implants. |
| FOR2107-Marburg and Muenster | Inclusion criteria: age 18-65 years; patients were diagnosed of bipolar I disorder by SCID- Interview, currently depressed, (hypo)manic or remitted. Exclusion criteria all: any MRI contraindications; any neurological abnormalities. Exclusion criteria controls: any current or former psychiatric disorder; Exclusion criteria patients: substance dependence or current benzodiazepine treatment (wash out of at least three half-lives before study participation) |
| MNC | Inclusion criteria: age 17-65 years; patients were diagnosed of bipolar I disorder by SCID-Interview, currently depressed (HAMD >= 18); Exclusion criteria all: any MRI contraindications; any neurological abnormalities; Exclusion criteria controls: any current or former psychiatric disorder; Exclusion criteria patients: substance-related disorders or current benzodiazepine treatment (wash out of at least three half-lives before study participation), and former electroconvulsive therapy |
| MilanoOSR | Exclusion criteria were age younger than 18; the presence of other diagnoses on Axis I; the presence of pregnancy; history of epilepsy or major medical, neurological disorders or brain trauma; history of drug or alcohol abuse or dependency. Patients were also required to have a current IQ in the normal range (>70). No patient had received electroconvulsive therapy within 6 months prior to study enrolment. |
| NUIG | Inclusion criteria: DSM-IV diagnosis of bipolar disorder (patients); age >18 and <60. Exclusion criteria: history of neurological illness (comorbid); lifetime DSM-IV axis 1 disorder or family history of psychotic or affective disorder in first- or second-degree relatives (controls); history of substance and/or alcohol misuse in the past year; learning disability; recent oral steroid use. |
| SBP | Patients: younger than 18 years, not in euthymic state. Controls: any psychiatric axis I or axis II disorder or neurological conditions, family history of schizophrenia or bipolar disorder in first-degree relatives, drug or alcohol abuse. |
| SCDS (IMHBD) | Exclusion criteria were age younger than 21 or older than 65 years, history of neurological disease or brain trauma, and alcohol/substance abuse in the 12 months prior to participation. All patients were diagnosed using DSM-IV, based on a detailed clinical interview and review of case notes. All healthy controls met the same exclusion criteria as the patients, and they were interviewed and excluded if they reported a history of mental illness and/or treatment with psychotropic medication other than non-regular use of benzodiazepines or similar drugs for insomnia. They were also questioned about family history of mental illness and excluded if a first-degree relative had experienced symptoms consistent with major psychiatric disorder and/or had received any form of in- or outpatient psychiatric care. The healthy controls were selected to be matched with the patients on demographic variables and on premorbid intelligence. |
| Sydney | Bipolar disorder participants meet DSM-IV criteria for either bipolar I or bipolar II disorder. Control participants meet criteria if no parent or sibling had bipolar I or II disorder, recurrent major depression, schizoaffective disorder, schizophrenia, recurrent substance abuse or any past psychiatric hospitalization; and no parent with a first degree relative had a past mood disorder hospitalization or history of psychosis. All subjects in are aged between 12 and 30 years. For those aged between 12 and 21 an adapted version of the Schedule for Affective Disorders and Schizophrenia for School-Age Children – Present and Lifetime Version (K-SADS-BP) was developed specifically for use in the US-Australia collaborative study of young people at genetic risk for BD. For participants aged between 22 and 30 the DIGS (Version 4) is used to measure the current and lifetime presence of axis I DSM-IV disorders. |
| Tulsa | Bipolar disorder participants met DSM-IV criteria for either bipolar I or bipolar II disorder, or BD NOS. Age 18-55. The unmedicated BD group did not receive any psychotropic medications for at least 3 weeks (8 for fluoxetine) prior to the MRI scanning. The healthy control individuals met the same exclusion criteria except that they had no personal or family (first-degree relatives) history of psychiatric illness assessed using the Structured Clinical Interview for the DSM-IV-TR and the Family Interview for Genetic Studies (FIGS). Exclusion criteria were as follows: serious suicidal ideation or behavior; medical conditions or concomitant medications likely to influence CNS or immunological function including cardiovascular, respiratory, endocrine and neurological diseases; a history of drug or alcohol abuse within 6 months or a history of drug or alcohol dependence within 1 year (DSM-IV-TR criteria), and general MRI exclusion criteria such as magnetic implants or claustrophobia. |
| UMCU | Inclusion criteria for all participants were: (1) age 18 years or older; (2) at least three Dutch-born grandparents; (3) a good understanding of Dutch language. Patients with a somatic illness that could have influenced the diagnosis of BD were excluded. Subjects with a history of head trauma or a neurological illness were excluded. Controls with a diagnosis of BD or a psychotic disorder or with a first-degree or second-degree relative with a diagnosis of BD or a psychotic disorder were excluded. |

**Table S5:** Image acquisition parameters and software versions used at each site

| Flip angle | 15 | 8 | 7 | 8 | 8 | 40 | 7 | 9 | 8 | 9 | 30 | 15 | 30 | 8 | 8 | 8 | 8 |
| --- | --- | --- | --- | --- | --- | --- | --- | --- | --- | --- | --- | --- | --- | --- | --- | --- | --- |
| TR (ms) | 2000 | 8.5 | 2530 | 4.76 | 9.76 | 25 | 8.4 | 1900 | 2130 | 7.4 | 25 | 1140 | 21 | 7.2 | 5.5 | 6 | 10 |
| TE (ms) | 3.93 | 3.046 | 1.53; 3.21; 4.89; 6.57 | 2.06 | 4.59 | 5 | 2.3 | 2.26 | 2.28 | 3.4 | 4.6 | 4.38 | 6 | 3.3 | 2.5 | 2.01 | 4.6 |
| TI (ms) | 710 | NA | 1100 | NA | NA | 0 | NA | 900 | 900 | 815 | NA | 600 | NA | 600 | NA | 725 | NA |
| Voxels (mm) | .47 x .47 x 1 | .83 x .83 x .89 | 1.3 x 1.0 x 1.3 | 1 x .6 x .6 | .859 x .859 x 1.2 mm | .9375 x .9375 x 1.5 | 1 x 1 x 1 | 1 x 1 x 1 | 1 x 1 x 1 | .5 x .5 x .5 | .9 x .9 x 1.6 acq; .9 x .9 x .8 rec | .45 x .45 x .9 | .7 x .7 x 1.8 | 1 x 1 x 1 | 1 x 1 x 1 | .938 x .0938 x .9 | .75×.75×.80 |
| Gap (mm) | 0 | 0 | 0 | 0 | 0 | 1.5 | 0 | 0.5 | 0 | 0 | 0.8 | 0 | 1.8 | 0 | 1 | 0 | 0 |
| Slices | 180 | 180 | 129 | 160 | 130 | 125 | 220 | 176 | 192 | 320 | 220 | 256 | 128 | 180 | 180 | 186 | 200 |
| Direction | Axial | Axial | Sagittal | Axial | Axial | Coronal | Sagittal | Sagittal | Sagittal | Coronal | Axial | Axial | Coronal | Axial | Sagittal | Axial | Sagittal |
| Sequence | 3D T1-weighted enhanced fast gradient echo (EFGRE3D) | 3T Philips Achieva Philips | 3D T1-weighted magnetization prepared rapid acquisition on gradient echo (MPRAGE) | 3D T1-weighted TFE | 3T Philips Intera | 3D T1-weighted spoiled gradient recalled acquisition in steady state | 3D T1-weighted turbo field echo (TFE) | 3D T1-weighted magnetization prepared rapid acquisition on gradient echo (MPRAGE) | 3D T1-weighted magnetization prepared rapid acquisition on gradient echo (MPRAGE) | 3D Fast gradient echo sequence | 3.0 Tesla scanner (Gyroscan Intera, Philips,) | 3D T1-weighted magnetization prepared rapid acquisition on gradient echo (MPRAGE) | 1.5 T Signa Excite | 3T Philips Achieva | 3D T1-weighted turbo field echo (TFE) | 3T GE MR750 Discovery | 3T Philips Achieva |
| MRI | 1.5T GE Signa | 3D T1-weighted MPRAGE | 3T Siemens Allegra | 3T Philips Achieva Philips | 3D ultrafast spoiled gradient echo sequence (SPGR) | 1.5T GE Signa | 3T Philips Achieva | 3T Siemens Magnetom Trio | 3T Siemens PRISMA | 3T Philips Gyroscan Intera | 3D T1-weighted enhanced fast field echo | 1.5T Siemens Magnetom | 3D‐SPGR | 3D T1-weighted magnetization prepared rapid acquisition gradient echo (MPRAGE) | 3T Philips Achieva | MPRAGE | 3D T1-weighted FFE |
| FreeSurfer | V5.3 | V5.3 | V5.3 | V5.3 | V5.3 | V5.3 | V5.1 | V5.3 | V5.3 | V5.3 | V6.0 | V5.3 | V5.1 | V5.3 | V5.3 | V6.0 | V5.1 |
| Site | Barcelona | CHRM2 | CIAM | Medellin (GIPSI) | Haarman (Moodinflame) | Halifax | Malt (Oslo) | FOR2107-Marburg | FOR2107-Muenster | MNC | MilanoOSR | NUIG | SBP | SCDS (IMHBD) | Sydney | Tulsa | UMCU |

**Table S6:** Number of participants removed from analysis in each region based on poor data quality or unreliable segmentation.

|  | Removed - n (%) | | |
| --- | --- | --- | --- |
|  | **Overall** | **BPD** | **Control** |
| Ventricles | 6 (0.25%) | 2 (0.08%) | 4 (0.16%) |
| Thalamus | 38 (1.56%) | 13 (0.53%) | 25 (1.03%) |
| Caudate nucleus | 32 (1.31%) | 10 (0.41%) | 22 (0.9%) |
| Putamen | 69 (2.83%) | 13 (0.53%) | 56 (2.3%) |
| Pallidum | 146 (5.99%) | 22 (0.9%) | 124 (5.09%) |
| Hippocampus | 34 (1.39%) | 17 (0.7%) | 17 (0.7%) |
| Amygdala | 40 (1.64%) | 11 (0.45%) | 29 (1.19%) |
| Nucleus Accumbens | 48 (1.97%) | 12 (0.49%) | 36 (1.48%) |

**Table S7:** Variance inflation factor (VIF) for each predictor in each model. Differences between models reflect the exclusion of different individuals in each.

| Model | Group | Age | Sex | Hemisphere | BMI | ICV |
| --- | --- | --- | --- | --- | --- | --- |
| Ventricles | 1.104 | 1.094 | 1.289 | 1.00 | 1.137 | 1.298 |
| Thalamus | 1.106 | 1.094 | 1.287 | 1.00 | 1.138 | 1.296 |
| Caudate nucleus | 1.103 | 1.092 | 1.286 | 1.00 | 1.137 | 1.294 |
| Putamen | 1.103 | 1.092 | 1.288 | 1.00 | 1.136 | 1.296 |
| Pallidum | 1.102 | 1.092 | 1.288 | 1.00 | 1.137 | 1.296 |
| Hippocampus | 1.105 | 1.095 | 1.288 | 1.00 | 1.138 | 1.297 |
| Amygdala | 1.105 | 1.093 | 1.289 | 1.00 | 1.137 | 1.298 |
| Nucleus accumbens | 1.104 | 1.093 | 1.29 | 1.00 | 1.136 | 1.298 |

**Table S8:** FDR-adjusted significance estimates for interactions between BMI and group (BPD vs. healthy control) in each region, with the AIC shown for variations of the model which exclude or include this interaction. Estimates of significance for the BMI by the number of medication classes, are shown for regions showing a significant effect of BMI.

|  |  |  | AIC | | |
| --- | --- | --- | --- | --- | --- |
| Region | **Interaction** | **Significance estimate** | **Excluding interaction** | **Including interaction** | **Improvement** |
| Lat. Ventricles | BMI by group | t(2412) = -1.41, p = .160 | 91819.3 | 91810.38 | 8.92 |
| Thalamus | BMI by group | t(2380) = 0.22, p = .829 | 74538.24 | 74534.94 | 3.3 |
| Caudate nucleus | BMI by group | t(2386) = -1.81, p = .071 | 67419.49 | 67413.9 | 5.59 |
| Putamen | BMI by group | t(2349) = -0.11, p = .913 | 70719.16 | 70716.23 | 2.93 |
| Pallidum | BMI by group | t(2272) = 1.58, p = .115 | 61168.42 | 61164.8 | 3.62 |
| Hippocampus | BMI by group | t(2384) = 0.85, p = .393 | 68366.57 | 68363.62 | 2.95 |
| Amygdala | BMI by group | t(2378) = 0.55, p = .580 | 62925.27 | 62924.19 | 1.08 |
| Nucleus accumbens | BMI by group | t(2370) = -0.59, p = .555 | 56604.2 | 56604.54 | -0.34 |
| Lat. Ventricles | BMI by medications | t(1097)= 0 .908, p = .364 | 42582.28 | 42574.79 | 7.49 |
| Amygdala | BMI by medications | t(1066) = 1.01, p = .314 | 41433.56 | 41426.09 | 7.47 |

**Table S9:** Association between clinical variables and BMI in patients when controlling for age, sex, and a random effect of data collection site

|  | R^2^ | Reference | Comparison | b | b SE | t | DF | p |
| --- | --- | --- | --- | --- | --- | --- | --- | --- |
| Number of medications (0-3) | 11.34% | *N/A* | *N/A* | 0.896 | 0.183 | 4.89 | 1100 | < 0.001 |
| Illness duration | 7.65% | *N/A* | *N/A* | 0.022 | 0.023 | 0.98 | 678 | 0.328 |
| History of psychotic symptoms | 9.06% | No | Yes | 0.078 | 0.514 | 0.15 | 488 | 0.879 |
| Lithium treatment | 6.03% | No | Yes | -0.167 | 0.399 | -0.42 | 736 | 0.676 |
| Diagnostic subtype | 5.89% | BD-I | BD-II | -0.153 | 0.458 | -0.33 | 795 | 0.738 |
|  |  | BD-I | BD-NOS | -1.288 | 2.382 | -0.54 | 795 | 0.589 |
|  |  | BD-II | BD-NOS | -1.134 | 2.386 | -0.47 | 795 | 0.635 |
| Mood state | 7.39% | Euthymic | Depressed | -0.354 | 0.768 | -0.46 | 610 | 0.645 |
|  |  | Euthymic | Hypomanic | -3.039 | 1.883 | 1.61 | 610 | 0.107 |
|  |  | Euthymic | Manic | 1.514 | 1.807 | 0.83 | 610 | 0.402 |
|  |  | Euthymic | Mixed | 1.609 | 3.764 | 0.42 | 610 | 0.669 |

**Figure S1:** BMI distribution of all participants


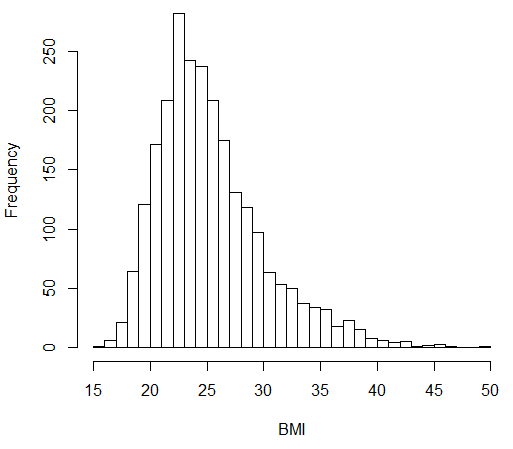


**Figure S2:** Linear vs. nonlinear BMI influence on volume

An important question was whether the influence of BMI on regional volume was linear or nonlinear. To investigate this, we categorized individuals within BMI categories: 1) 15-19.9, 2) 20-24.9, 3) 25-29.9, or 4) 30-34.9. Participants were scored according to their relative BMI within categories, ranging from +0 to +4.9. A nonlinear association between BMI and ventricular volume would manifest as differences between BMI categories in terms of the regression slope for within-category predictions of ventricular volume. This was tested using an interaction between BMI category and relative BMI within categories. This interaction is shown in Figure S3, where each category demonstrated a larger ventricular volume than the last, but similar slopes. This interaction was not significant (t(2261) = .116, p = .907), suggesting that the effect of BMI on ventricular volume is approximately linear. Furthermore, when comparing two identical models, one using BMI and one using BMI^2^, the former has a better AIC (90697 vs. 90705), a better BIC (90787 vs. 90796), and a considerably higher goodness of fit (R^2^ = 0.917 vs. R^2^ = 0.279). While this interaction is similarly not significant in the amygdala (t(2228) = 1.12, p = .263), the slopes are less similar between groups for this region, suggesting some potential nonlinearity that may not be accountable in this sample. As with the ventricles, fitting BMI rather than BMI^2^ results in a better AIC (62140 vs. 62148), BIC (62230 vs. 62239), and goodness of fit (R^2^ = .926 vs. R^2^ = .193).


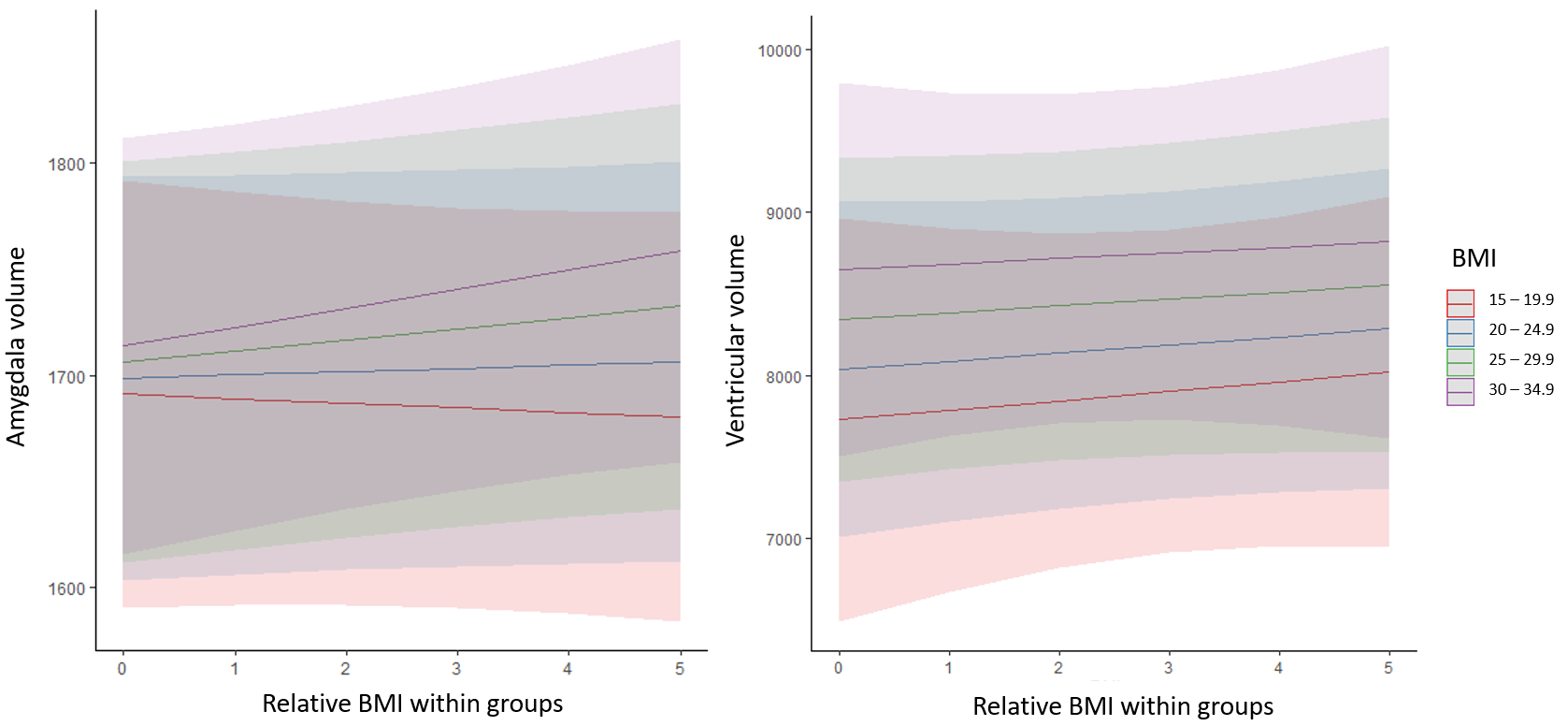


**Figure S2** The interaction between BMI categories and within-category relative BMI in predicting ventricular volume, demonstrating equivalent slopes at all BMI ranges, for an approximately linear relationship.

**Figure S3:** Examining inter-site differences

While we controlled for a random effect of data collection site in all models, and this step was intended to account for any inter-site variability in regional volumes that were unrelated to our predictors, it was still possible that differences between site in diagnostic or recruitment criteria may have confounded our association between BMI and volume. To investigate this, we additionally modeled an interaction between BMI and site in each of the regions where a significant association existed between BMI and volume (the lateral ventricles and amygdala), while controlling for main effects (group, BMI), covariates (age, sex, hemisphere) and all random effects. The BMI by site interaction was not significant in either the lateral ventricles ((F(13,2370)=1.265, p=.23) or amygdala (F(13,2336)=1.677, p=.06). That is, the association between BMI and volume was statistically equivalent between sites, regardless of specific sources of potential variation, in both regions. These findings suggest that any variability between sites did not influence the association between BMI and volume to a significant degree.


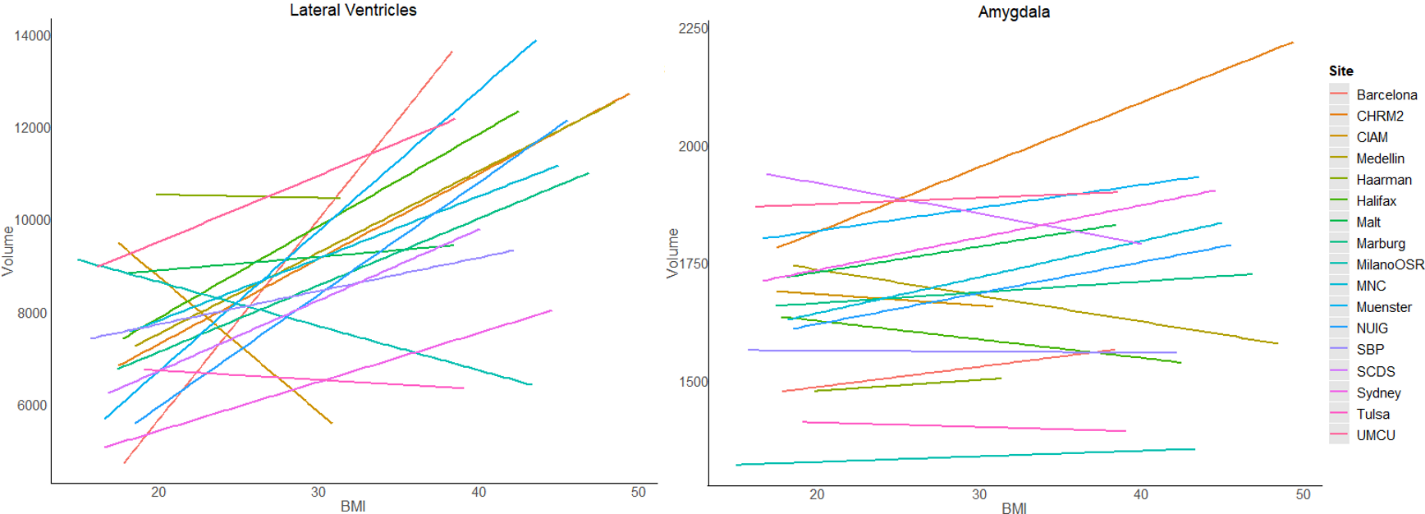


**Figure S3** Regression slopes for BMI in all sites, modeled using a BMI by site interaction, in each of the regions where BMI showed a significant main effect. This interaction was non-significant in both the lateral ventricles and amygdala, showing generally positive associations between BMI and volume.

***Supplemental Methods***

***Description of fixed and random effects in each model***

Model specifications for each analysis type are outlined below, in *R* syntax for regression models. In each case, *Volume* refers to the subcortical volume (mm^3^) of the region being modeled, *Dx* refers to the categorical grouping of participants (BPD or healthy control), and *ICV* (total intracranial volume in mm^3^) was used to control for overall volume differences between individuals. In the analysis of drugs, *NumMeds* refers to the number of categories of medication prescribed to an individual at the time of data collection, random from zero to four. *Hemisphere* (left or right) controlled for inter-hemisphere volume differences for a region, both overall (fixed effect) and within individuals (random effect), allowing for estimation of a region bilaterally using a single model, for a total of 8 models (one for each region). In addition to the fixed effects specified below, every model used the same random effect structure (1 + hemisphere | Site / Subject), allowing for random variability both between individuals and between sites overall.

**Modeling regional volume**

1. *Volume = Dx + Age + Sex + hemisphere + ICV*
2. *Volume = BMI + Age + Sex + hemisphere + ICV*
3. *Volume = Dx + BMI + Age + Sex + hemisphere + ICV*

**Mediation**

1. Direct effect (*Dx > Volume*, without *BMI*): *Volume = Dx + age + sex + hemisphere + ICV*
2. Indirect effect (a, *Dx > BM*I): *BMI = Dx + age + sex + hemisphere*
3. Total effect (*Dx > volume*, with *BMI*): *Volume ~ Dx + BMI + age + sex + hemisphere + ICV*

**Drugs**

1. *Volume = NumMeds + age + sex + hemisphere + ICV*
2. *Volume = BMI + age + sex + hemisphere + ICV*
3. *Volume = NumMeds + BMI + age + sex + hemisphere + ICV*
4. *Volume = Lithium(Y/N) + BMI + age + sex + hemisphere + ICV*
